# Enhancing HDAC Inhibitor Screening: Addressing Zinc Parameterization and Ligand Protonation in Docking Studies

**DOI:** 10.3390/ijms26020850

**Published:** 2025-01-20

**Authors:** Rocco Buccheri, Alessandro Coco, Lorella Pasquinucci, Emanuele Amata, Agostino Marrazzo, Antonio Rescifina

**Affiliations:** Department of Drug and Health Sciences, University of Catania, Viale A. Doria 6, 95125 Catania, Italy; rocco.buccheri@studium.unict.it (R.B.); alessandro.coco@phd.unict.it (A.C.); lpasquin@unict.it (L.P.); eamata@unict.it (E.A.); marrazzo@unict.it (A.M.)

**Keywords:** HDAC inhibitors, hydroxamic acid ligands, metalloprotein docking, zinc-binding interactions, free energy of binding prediction, molecular docking algorithms, structure-based drug design

## Abstract

Precise binding free-energy predictions for ligands targeting metalloproteins, especially zinc-containing histone deacetylase (HDAC) enzymes, require specialized computational approaches due to the unique interactions at metal-binding sites. This study evaluates a docking algorithm optimized for zinc coordination to determine whether it could accurately differentiate between protonated and deprotonated states of hydroxamic acid ligands, a key functional group in HDAC inhibitors (HDACi). By systematically analyzing both protonation states, we sought to identify which state produces docking poses and binding energy estimates most closely aligned with experimental values. The docking algorithm was applied across HDAC 2, 4, and 8, comparing protonated and deprotonated ligand correlations to experimental data. The results demonstrate that the deprotonated state consistently yielded stronger correlations with experimental data, with R^2^ values for deprotonated ligands outperforming protonated counterparts in all HDAC targets (average R^2^ = 0.80 compared to the protonated form where R^2^ = 0.67). These findings emphasize the significance of proper ligand protonation in molecular docking studies of zinc-binding enzymes, particularly HDACs, and suggest that deprotonation enhances predictive accuracy. The study’s methodology provides a robust foundation for improved virtual screening protocols to evaluate large ligand libraries efficiently. This approach supports the streamlined discovery of high-affinity, zinc-binding HDACi, advancing therapeutic exploration of metalloprotein targets. A comprehensive, step-by-step tutorial is provided to facilitate a thorough understanding of the methodology and enable reproducibility of the results.

## 1. Introduction

Histone deacetylases (HDACs) are crucial in the epigenetic regulation of DNA replication and transcription. They act on histone proteins within the cell nucleus, contributing to nucleosome formation and regulating chromatin folding and extension. HDACs remove acetyl groups from lysine residues on histone proteins, promoting chromatin compaction, while histone acetyltransferases (HATs) acetylate lysine residues, leading to chromatin relaxation. Chromatin extension makes DNA accessible to enzymes involved in replication and transcription, whereas chromatin folding restricts access to these enzymes [1].

Histone deacetylase HDAC involvement has been linked to numerous diseases, suggesting that HDAC inhibitors (HDACi) could be explored as potential antitumor and neuroprotective agents. Recent studies have shown HDAC involvement in neurodegenerative disorders, such as Alzheimer’s disease [2], as well as in various cancers [3].

The search for effective HDACi has led to the development and commercialization of drugs like Belinostat, Panobinostat, and Vorinostat [4], all of which contain the hydroxamic acid functional group, a well-recognized zinc-binding group (ZBG) [5].

This underscores the need to optimize molecular docking protocols to enhance the identification of potentially active HDACi, particularly during virtual screening efforts. However, accurately predicting the binding free energies and poses of HDACi has been challenging due to the involvement of zinc ions, which are essential for the catalytic activity of HDACs. As a divalent metal cation, zinc has unique coordination and electrostatic properties that can complicate docking and scoring procedures. One of the main challenges with docking software is the inadequate parameterization of zinc ions, which are often treated as simple divalent cations. This ignores zinc’s ability to form coordination complexes.

Accurately determining binding free energies for ligands targeting metalloproteins, particularly those containing zinc ions, necessitates specialized computational approaches [6]. Standard docking programs often fail to model metal center interactions effectively, leading to inaccurate predictions. To address this limitation, advanced computational tools and methods have been developed specifically to model metalloproteins, focusing on zinc coordination environments. Examples of these tools, listed in chronological order of implementation, include FlexX [7,8,9,10], AutoDockZn [11], MpsDockZn [12], GM-DockZn [13], Autodock-Vina [14,15], GPDOCK [16], AutoDock Bias [17] (a modification within the AutoDock suite), and MetalDock [18].

For zinc-containing HDAC enzymes, the following methodology can be employed to enhance docking accuracy and binding energy calculations: (i) preprocessing the zinc coordination sphere by explicitly modeling its coordination geometry (typically tetrahedral) to ensure accurate geometry during docking setup. This includes specifying coordination partners such as histidine, aspartate, water molecules, and potential ligand interactions with the zinc ion. (ii) Precise ligand parameterization is essential, given that accurate protonation states and charge distribution are crucial for HDACi, particularly for functional groups like hydroxamates or carboxylates, which interact with zinc.

We employed the AutoDock Bias tool to implement the first step, which introduces scoring biases favoring hydrogen bond donors near the zinc ion, thereby better mimicking zinc coordination bonds. Recently, the authors proposed an advanced approach known as “metalloprotein bias docking” (MBD), which extends the AutoDock Bias method [19]. MBD has shown superior accuracy in predicting poses and ligand-binding free energies, although this approach has yet to be applied explicitly to HDACs.

For the second step, we assessed whether to parameterize hydroxamic acids in their *O*-undissociated or *O*-dissociated forms, given that most studies use the undissociated state [6,20]. Since 2007, several authors have demonstrated, through DFT computational studies, that hydroxamic acid tends to be deprotonated within the active site [21,22,23]. However, to the best of our knowledge, only a few studies, primarily by the Sippl group, have specifically investigated this dissociated form. These studies provide limited explanation beyond its potential to more accurately reproduce the native bidentate chelation poses observed in co-crystallized ligand structures [24,25,26,27,28,29,30].

**Figure 1:**
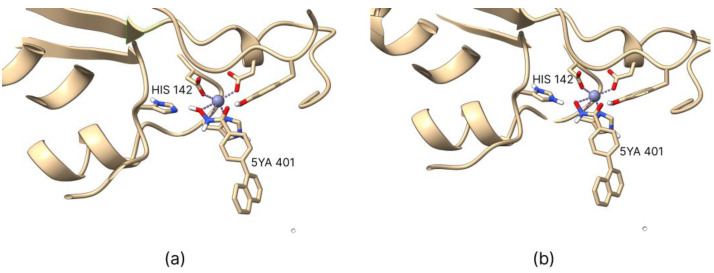
The protonation states of the histidine residues of the histone deacetylase (HDAC) 8 enzyme remained unchanged from the PDB file for the protonated ligand series (**a**), with His142 protonated in the deprotonated ligand series (**b**). Images were generated using UCSF ChimeraX software, and the co-crystallized hydroxamic ligand 5YA 401 was shown as a reference.

Considering that HDAC isoforms typically exhibit conserved interactions near the zinc-binding site, involving histidine and tyrosine residues that stabilize the ZBG through hydrogen bonds [31], we examined two possible protonation states, shown in Figure 1 for HDAC 8.

We focused on the two histidines adjacent to the hydroxamic acid moiety, hypothesizing that the deprotonated form would exhibit stronger metal ion chelation due to increased negative charge density on the hydroxamic oxygen, facilitated by the histidine residues in proximity [32]. The primary objective of this study was to evaluate whether a docking algorithm optimized for zinc interactions could distinguish between protonated and deprotonated hydroxamic acid ligands, identifying the best poses and calculated binding energies to match experimental values closely. This evaluation would support rapid virtual screening for large ligand libraries, optimizing expected results.

## 2. Results and Discussion

### 2.1. Coordination Geometry of the Zinc Ion

A notable limitation of the AutoDock Bias algorithm is its inability to assign biases to the zinc ion due to the complex nature of its coordination bonds, which cannot be generalized and must be assessed individually for each protein. To address this, the coordination geometry of the zinc ion within HDAC enzymes was studied using co-crystallized PDB structures containing hydroxamic acid as a co-crystallized ligand. The analysis revealed that the coordination geometry is consistently maintained across all examined PDB entries, with zinc ions coordinated by two histidine residues and one aspartate residue. Based on this observation, a Python script was developed to predict the ideal positions of hydrogen bond acceptor groups in the ligand, using the known coordination geometry once the co-crystallized ligand is removed.

**Figure 2:**
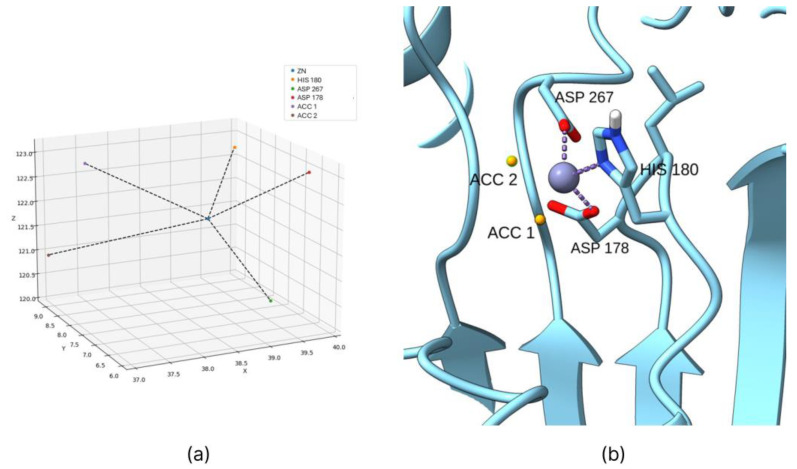
The coordination bonds predicted for HDAC 8 enzyme by the Python script (**a**) and the actual positions introduced as biases ((**b**), generated in UCSF ChimeraX) are represented by yellow spheres.

This approach accurately predicts the ideal interaction positions of hydroxamic ligands, enabling the calculation of bias positions for each PDB structure, even when hydroxamic acid is not present as the co-crystallized ligand (Figure 2).

### 2.2. Active Ligands Selection

To ensure biological consistency, active ligands for each HDAC isoform were selected from the same study [21]. Importantly, all the activity values, expressed as *K*_i_, were derived using the same binding assay, facilitating accurate comparison across datasets. Using activity data from different and/or biologically incompatible assays could compromise experimental integrity, potentially skewing results and undermining the reliability of subsequent docking studies. The selection of the isoforms for this study was guided by the necessity to include a common ligand across all cases examined while ensuring that the *K*_i_ values spanned a range up to a maximum of 10 μM. This approach was adopted to enhance the statistical reliability of the data analysis. Consequently, isoforms 2, 4, and 8 and the ligands listed in Table 1 were chosen. The in silico obtained *K*_i_ values, calculated as described in Section 2.4, have been included in the same table for easy comparison.

### 2.3. Introduction of Biases

Biases were introduced after thoroughly analyzing the binding site for each HDAC isoform. The most critical interactions between the co-crystallized ligand and the target protein were identified, and specific biases related to the zinc atom were applied to these interactions. One significant limitation of AutoDock Bias is its inability to parameterize the zinc atom accurately. To overcome this, a Python script was developed to predict the biases to be applied for zinc ion interactions within HDAC enzymes.

The correct interaction geometry between zinc ions and hydroxamic acids was studied using multiple co-crystallized structures containing hydroxamic acids and ligands. These studies confirmed that the zinc coordination geometry remains consistent across various co-crystals, with the zinc ion always coordinated by one histidine residue and two aspartate residues. Among the aspartate residues, one typically positions slightly further from the zinc ion than the other. The Python script takes this coordination geometry into account, calculating the ideal interaction coordinates between the hydroxamic ligand and the zinc ion based on the relative positions of the histidine and aspartate residues in the PDB structure (with the ligand removed).

**Figure 3:**
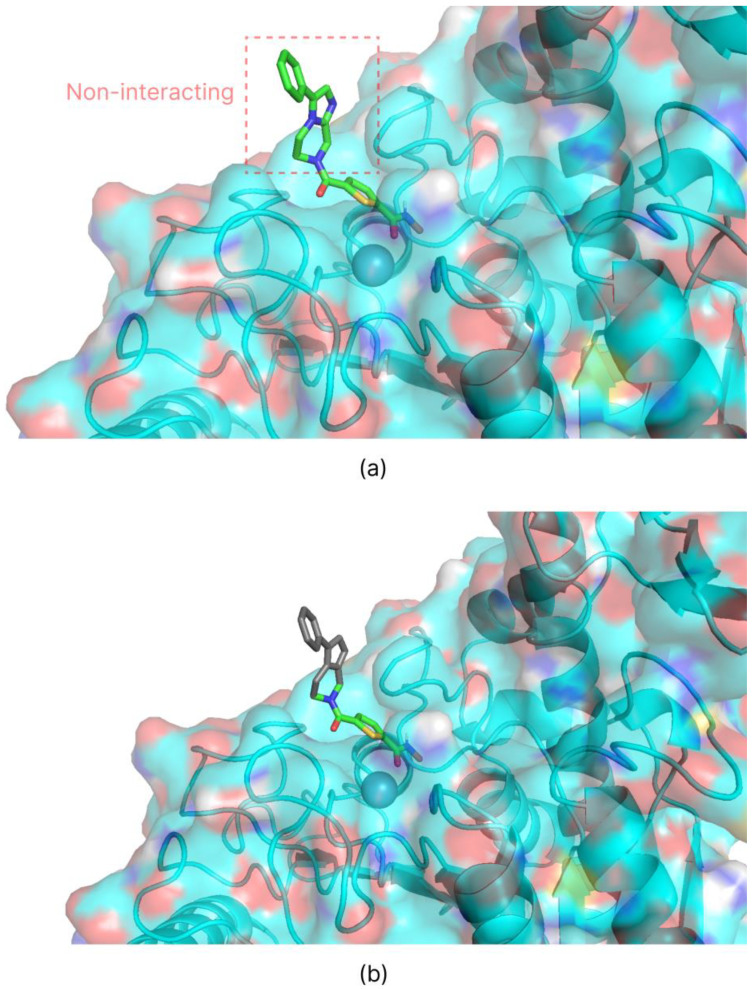
Graphical visualization obtained by PyMOL Molecular Graphics System (v. 3.1.0, Schrödinger, L., & DeLano, W. (2020). PyMOL. Retrieved from http://www.pymol.org/pymol) of the portion of the co-crystallized ligand of HDAC 4 not interacting with the calpain site (**a**). For the RMSD calculation, the gray portion (**b**) was not considered.

Root mean square deviation (RMSD) studies were performed using the methods described in the AutoDock Bias documentation to confirm the validity of the introduced biases. Re-docking of the co-crystallized ligand was performed first without and then with the introduction of the biases. Biases that maintained unchanged or improved the RMSD value were considered valid. For the RMSD analysis of the HDAC 4 isoform, only the portion of the ligand that interacts with the target (Figure 3b) was considered, neglecting the part that escapes from the catalytic site (Figure 3a). Since the latter has no intermolecular interactions with the protein’s amino acids, it was considered insignificant to the evaluation file.

The results presented in Table 2 underscore the validity of introducing biases, as all RMSD values obtained with the applied biases fall within the widely accepted threshold of 3 Å [33], typically indicative of reliable RMSD values. Specifically, the analyses performed for HDAC isoforms 4 and 8 validate the effectiveness of the re-docking process for their co-crystallized ligands. For HDAC isoform 2, the introduction of biases significantly enhances docking accuracy.

Detailed analysis of the poses generated for HDAC isoform 2 highlights that, in the absence of biases, the ligand coordinates the zinc ion via the amide group, deviating from the interaction observed in the co-crystal structure. However, introducing a bias toward the zinc ion dramatically improves the pose generation, aligning the results closely with the co-crystal structure. This finding is crucial as it enhances the docking algorithm’s precision in re-docking scenarios and substantiates a model otherwise considered inadequate for molecular docking applications.

The Python script, a tutorial on predicting the bias positions for zinc ions in HDAC enzymes, grid position coordinates, biases coordinates, and all relevant virtual screening (VS) files are available in our GitHub repository (see Supplementary Materials).

### 2.4. Virtual Screening

VSs of ligand sets prepared in both protonated and deprotonated forms were performed. The free energy of binding (Δ*G*) associated with the first pose was used to evaluate the interaction of each ligand. To ensure reproducibility, energy values were extracted from the output files by considering the “Mean Binding Energy” of the most populated cluster as the representative energy value. In cases where clusters had similar populations, the free energy of binding was averaged across these clusters.

The derived Δ*G* value was then converted to the corresponding *K*_i_ value using the thermodynamic relationship:

*K*_i_ = e^(Δ*G*/RT)^ (1)

The equation was solved at T = 310 K, with R = 0.0019872036 kcal/(K × mol). Subsequently, the *K*_i_ values were transformed into their negative logarithm, p*K*_i_.

The so calculated p*K*_i_ values were then compared with experimental p*K*_i_ values (Table 1) via a scatter plot, and regression analysis was conducted to obtain the correlation coefficient (R^2^), which was used to evaluate and compare the results.

**Figure 4:**
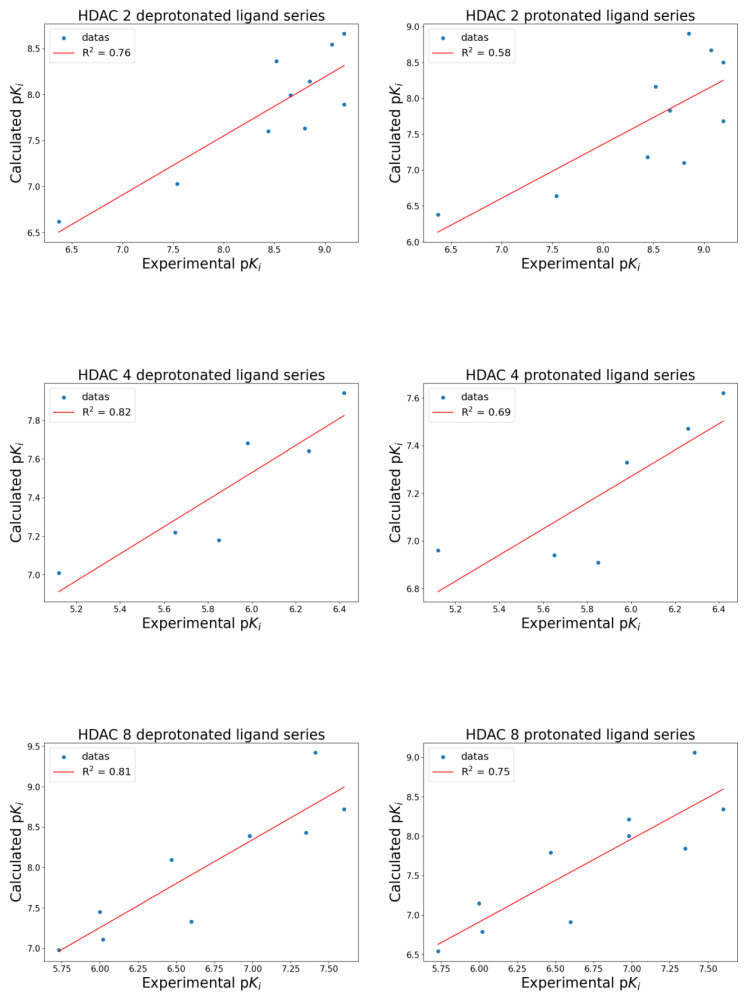
Correlation graphs of HDAC 2 (**top**), HDAC 4 (**middle**), and HDAC 8 (**bottom**). Graphs show the data distribution (blue dots) and the linear regression line (red line) of correlations between experimental and calculated p*K*_i_ for the deprotonated ligand series (**left**) and the protonated ligand series (**right**).

The docking results (Figure 4) indicate that deprotonated hydroxamic ligands correlate better with experimental data than protonated ligands. The correlation between deprotonated ligands and experimental data consistently outperformed protonated ligands.

Specifically, the R^2^ for deprotonated versus protonated ligands were as follows: for HDAC 2, R^2^_deprotonates_ = 0.76 and R^2^_protonates_ = 0.58; for HDAC 4, R^2^_deprotonates_ = 0.82 and R^2^_protonates_ = 0.69; for HDAC 8, R^2^_deprotonates_ = 0.81 and R^2^_protonates_ = 0.75.

**Figure 5:**
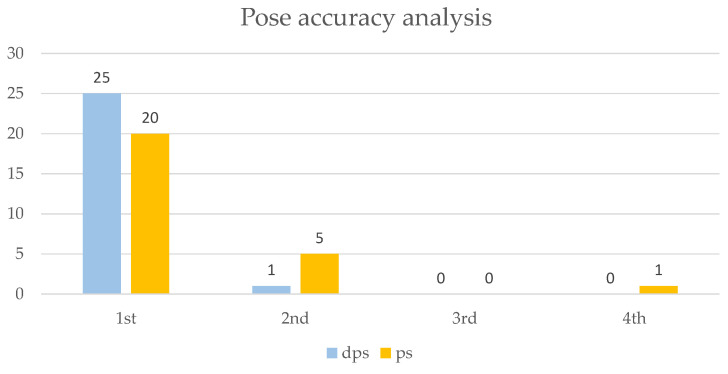
Graphical representation of the distribution of poses in the relative positions by cumulatively evaluating the poses of all targets. The distributions for the deprotonated ligand series (dps, light blue) and the protonated ligand series (ps, yellow) are shown.

In addition, the accuracy of the poses generated by the docking algorithm was analyzed, focusing on the ability to correctly position the hydroxamic group of the ligand to coordinate the zinc ion. Each target’s selected poses (Tables S1 and S2) were considered. The chosen poses were those in which the ligand’s hydroxamic group demonstrated coordination binding with the zinc ion. A total of 26 poses were analyzed across the protonated and deprotonated ligand series. The number of instances in which the corrected pose, with proper coordination of the hydroxamic group, appeared in the first, second, third, or fourth position in the docking results was recorded to evaluate pose accuracy. Percent pose accuracy was calculated as the ratio of the instances in which the correct pose ranked first to the total poses analyzed. As illustrated in Figure 5, deprotonated ligands displayed a significantly higher accuracy, with the hydroxamic group correctly positioned in the first pose in 96.2% of cases, compared to only 76.9% for protonated ligands. This represents a 25.1% improvement in the prediction of correct poses when using deprotonated ligands, highlighting their enhanced suitability for accurate docking predictions.

Two key findings emerge from this study. The most significant result is the comparison between protonated and deprotonated ligands. As predicted theoretically, the interaction between the zinc ion and the electron lone pairs is more favorable when the electron density on the electronegative atom is higher, as in the case of the deprotonated ligand. Experimental results strongly support this theoretical assumption. Specifically, the docking algorithm generated energy values that correlated better with experimental data for the deprotonated ligand series than the protonated ones. These findings underscore two crucial factors for enhancing the accuracy of Δ*G* of binding predictions in metalloprotein docking, particularly for HDACs. First, ligands should be deprotonated, and the histidine residues in the proteins should be protonated. Second, the custom zinc coordination Python script we developed for AutoDock Bias should be used when working with HDACs to ensure the zinc interaction is appropriately accounted for by the docking algorithm.

The second noteworthy result is comparing the quality of the poses obtained. By using hydroxamic ligands in the deprotonated form, the docking algorithm can more easily identify the group coordinating the zinc ion, almost always positioning it as the best result. This further supports the conclusion that deprotonated ligands are preferable, showing greater consistency with experimental data.

## 3. Materials and Methods

### 3.1. Biological Data and 3D Structures Generation

The chemical structures of ligands active on HDAC enzymes were selected from a single source to ensure biological consistency across binding assays. Hydroxamic-acid-containing ligands were prioritized, and their affinity values (*K*_i_) were converted from µM to nM, subsequently expressed as decimal negative logarithms (p*K*_i_ = −log*K*_i_). Ligand preparation was conducted first using RDKit open-source toolkit for cheminformatics (v. 2023.09.6) using the Python script rdconf.py download from GitHub repository “rdkit-scripts” (https://github.com/dkoes/rdkit-scripts, accessed on 12 March 2024), through which SMILES codes were used to generate 3D structures in single conformer and using the ETKDG knowledge-based method [34] instead of distance geometry.

Ligand preparation continued in Open Babel (v. 3.1.1) [35], where sdf format was converted into pdbqt format for compatibility with AutoDock4 [36], reflecting physiological pH (7.4) states. For *O*-deprotonated ligands, the hydroxamic OH hydrogen atom was manually removed before 3D structure generation, after which structures were optimized as outlined.

### 3.2. Protein Preparation

The protein structures were downloaded from the Protein Data Bank (https://www.rcsb.org/, accessed on 3 March 2024). Due to their significant pharmaceutical importance, human-HDAC isoforms 2, 4, and 8 (PDB IDs: 4LXZ, 2VQM, and 5FCW, respectively) were chosen. Chain A was selected for analysis for each protein structure.

Proteins were prepared for docking using the UCSF ChimeraX software (v. 1.7.1, University of California, San Francisco, CA, USA) [37]. Non-relevant domains, water molecules, and other non-functional co-crystallized molecules for docking were removed. The preparation of the proteins in the presence of the co-crystallized ligand was carried out using the integrated dockprep option in the software, which performs protonation at physiological pH, amino acid discretization according to appropriate rotamers, and assignment of charges to metal ions (in our case, Zn^2+^).

The prepared structures were minimized using the YASARA software (v. 23.12.24, YASARA Biosciences GmbH, Vienna, Austria), force field AMBER14 was applied, and minimization was carried out according to the default settings via the Energy Minimization option.

Subsequently, the ligand was removed, and the protein’s three-dimensional structure was saved in pdb format compatible with the AutoDock4 algorithm.

To docking ligands in the deprotonated hydroxamate form, the above structures of each HDAC isoform were generated with the His145 (HDAC 2), His158 (HDAC 4), and His142 (HDAC 8) in the protonated HIP form.

### 3.3. Molecular Docking Simulation

Virtual screening was performed through the AutoDock Bias suite integrated within the AutoDock Tools (v. 1.5.7). The algorithm employed was AutoDock4 (v. 4.2.6) with the Lamarckian Genetic Algorithm (LGA). Through AutoGrid4 (v. 4.2.6), the grid was centered with the co-crystallized ligand, and its size was extended by 70 grid points in three dimensions of space. Docking was performed with the GA parameters configured as follows: ga_runs = 100, ga_pop_size = 150, ga_num_evals = 2,500,000, ga_num_generations = 27,000, ga_elitism = 1, ga_mutation_rate = 0.02, ga_crossover_rate = 0.8, ga_crossover_mode = two points, ga_cauchy_alpha = 0.0, ga_cauchy_beta = 1.0, number of generations for picking worst individual = 10.

Before docking, all the scripts given in AutoDock Bias’s ‘User guide’ were used to prepare the ligand, receptor, AutoGrid Grid Parameter File, and all docking parameter files. Grid position coordinates used for docking and bias coordinates applied to each target are reported in Tables S3 and S4.

## 4. Conclusions

In conclusion, this study evaluates the efficacy of a zinc-optimized docking algorithm in distinguishing between protonated and deprotonated states of hydroxamic acid ligands when targeting HDAC enzymes. By rigorously analyzing both protonation states, we aimed to determine which form yielded docking poses and binding energy predictions that more closely aligned with experimental data. Our findings indicate that deprotonated ligands consistently demonstrated a stronger correlation with experimental results across all HDAC targets. Specifically, for HDAC 2, 4, and 8, deprotonated ligands showed superior R^2^ values, highlighting the enhanced accuracy of docking predictions with the deprotonated form.

This outcome underscores the importance of considering ligand protonation states in molecular docking studies of zinc-containing enzymes, as using deprotonated hydroxamic groups in silico offers docking energy values that more reliably mirror experimental outcomes. Ultimately, these findings provide a foundation for improved computational workflows, facilitating rapid virtual screening for HDACi and other metalloprotein-targeting drugs, thereby streamlining the discovery of high-affinity, zinc-binding ligands with significant therapeutic potential.

## Figures and Tables

**Table 1:** Experimental and calculated p*K*_i_ values of examined ligands containing the hydroxamic group.

Isoform	Name ^1^	Experimental *K*_i_ (nM)	Experimental p*K*_i_	Calculated p*K*_i_ dps ^2^	Calculated p*K*_i_ ps ^3^
HDAC 2	LBH-589	0.65	9.19	8.66	8.50
	Trichostatin A	0.65	9.19	7.89	7.68
	PXD-101	0.85	9.07	8.54	8.67
	LAQ-824	1.40	8.85	8.14	8.90
	SAHA	1.60	8.80	7.63	7.10
	Scriptaid	2.20	8.66	7.99	7.83
	ITF-2357	3.00	8.52	8.36	8.16
	Pyroxamide	3.60	8.44	7.60	7.18
	SHA	29.00	7.54	7.03	6.64
	4-PBHA	430.00	6.37	6.62	6.38
HDAC 4	PXD-101	380.00	6.42	7.94	7.62
	LBH-589	550.00	6.26	7.64	7.47
	ITF-2357	1050.00	5.98	7.68	7.33
	Trichostatin A	1400.00	5.85	7.18	6.91
	LAQ-824	2250.00	5.65	7.22	6.94
	Scriptaid	7500.00	5.12	7.01	6.96
HDAC 8	PXD-101	25.00	7.60	8.72	8.34
	ITF-2357	39.00	7.41	9.42	9.06
	Trichostatin A	45.00	7.35	8.43	7.84
	LBH-589	105.00	6.98	8.39	8.00
	Scriptaid	105.00	6.98	8.39	8.21
	SAHA	250.00	6.60	7.33	6.91
	LAQ-824	340.00	6.47	8.09	7.79
	SHA	950.00	6.02	7.11	6.79
	Pyroxamide	1000.00	6.00	7.45	7.15
	4-PBHA	1850.00	5.73	6.98	6.54

^1^ The color indicates the ligand’s presence across all three isoforms. ^2^ dps refers to the deprotonated series. ^3^ ps refers to the protonated series.

**Table 2:** RMSD values calculated for co-crystallized ligand re-docking analyses performed without and with the introduction of bias.

Isoform	Nonbiased RMSD (Å)	Biased RMSD (Å)
HDAC 2	8.28	1.98
HDAC 4	1.67	1.66
HDAC 8	2.91	2.93

## Data Availability

All data generated or analyzed during this study are included in this published article and available on GitHub: https://github.com/rocco-b/Zinc_Package (accessed on 6 December 2024).

## Supplementary Materials

The supporting information can be downloaded at: https://www.mdpi.com/article/10.3390/ijms26020850/s1.
