# Supplementary material for: Enhancing HDAC Inhibitor Screening: Addressing Zinc Parameterization and Ligand Protonation in Docking Studies
Source: Int J Mol Sci. 2025 Jan 20;26(2):850. doi: 10.3390/ijms26020850 (PMC11766394; doi:10.3390/ijms26020850)
Supplement: Supplementary file 1 [file ijms-26-00850-s001.zip › ijms-3410250-supplementary.pdf]

International Journal of
Molecular Sciences

MDPI

Supplementary Materials

Enhancing HDAC Inhibitors Screening: Addressing Zinc Parameterization and Ligand Protonation in Docking Studies

Rocco Buccheri †, Alessandro Coco †, Lorella Pasquinucci, Emanuele Amata, Agostino Marrazzo and Antonio Rescifina*

Department of Drug and Health Sciences, University of Catania, Viale A. Doria 6, 95125 Catania, Italy; rocco.buccheri@studium.unict.it (R.B.), alessandro.coco@phd.unict.it (A.C.), lpasquin@unict.it (L.P.), eamata@unict.it (E.A.), marrazzo@unict.it (A.M.)

* Correspondence: arescifina@unict.it

† These authors contributed equally

Table S1. Complete docking data for deprotonated ligand series. The Δ*G* values shown were derived by considering the Mean Binding Energy of the first ligand pose in which the hydroxamic functional group coordinates the zinc ion.

Protein	Ligand	Δ*G* (kcal/mol)	K_i (nM)	pK_i	Pose
HDAC 2	LBH-589	−12.29	2.17	8.66	1
	Trichostatin A	−11.19	12.92	7.89	1
	PXD-101	−12.11	2.90	8.54	1
	LAQ-824	−11.55	7.20	8.14	1
	SAHA	−10.82	23.55	7.63	1
	Scriptaid	−11.34	10.13	7.99	1
	ITF-2357	−11.86	4.35	8.36	1
	Pyroxamide	−10.78	25.13	7.60	1
	SHA	−9.97	93.60	7.03	1
	4-PBHA	−9.39	239.99	6.62	1
HDAC 4	PXD-101	−11.26	11.53	7.94	2
	LBH-589	−10.84	22.80	7.64	1
	ITF-2357	−10.90	20.69	7.68	1
	Trichostatin A	−10.18	66.57	7.18	1
	LAQ-824	−10.24	60.39	7.22	1
	Scriptaid	−9.95	96.69	7.01	1
HDAC 8	PXD-101	−12.37	1.90	8.72	1
	ITF-2357	−13.36	0.38	9.42	1
	Trichostatin A	−11.96	3.70	8.43	1
	LBH-589	−11.9	4.08	8.39	1
	Scriptaid	−11.9	4.08	8.39	1
	SAHA	−10.4	46.57	7.33	1
	LAQ-824	−11.48	8.07	8.09	1
	SHA	−10.08	78.30	7.11	1
	Pyroxamide	−10.57	35.34	7.45	1
	4-PBHA	−9.9	104.87	6.98	1

Table S2. Complete docking data for protonated ligand series. The ΔG values shown were derived by considering the Mean Binding Energy of the first ligand pose in which the hydroxamic functional group coordinates the zinc ion.

Protein	Ligand	ΔG (kcal/mol)	K_i (nM)	pK_i	Pose
HDAC 2	LBH-589	−12.05	3.20	8.50	1
	Trichostatin A	−10.89	21.02	7.68	1
	PXD-101	−12.3	2.13	8.67	1
	LAQ-824	−12.62	1.27	8.90	1
	SAHA	−10.07	79.58	7.10	1
	Scriptaid	−11.1	14.95	7.83	1
	ITF-2357	−11.58	6.86	8.16	1
	Pyroxamide	−10.18	66.57	7.18	2
	SHA	−9.42	228.58	6.64	1
	4-PBHA	−9.05	416.75	6.38	1
HDAC 4	PXD-101	−10.81	23.94	7.62	1
	LBH-589	−10.59	34.21	7.47	1
	ITF-2357	−10.40	46.57	7.33	2
	Trichostatin A	−9.80	123.35	6.91	1
	LAQ-824	−9.85	113.73	6.94	4
	Scriptaid	−9.87	110.10	6.96	2
HDAC 8	PXD-101	−11.83	4.57	8.34	1
	ITF-2357	−12.85	0.87	9.06	1
	Trichostatin A	−11.12	14.47	7.84	1
	LBH-589	−11.35	9.96	8.00	1
	Scriptaid	−11.65	6.12	8.21	1
	SAHA	−9.8	123.35	6.91	2
	LAQ-824	−11.05	16.21	7.79	1
	SHA	−9.63	162.55	6.79	2
	Pyroxamide	−10.14	71.03	7.15	1
	4-PBHA	−9.27	291.60	6.54	1

Table S3. Grid position coordinates used for docking. For all targets, grid spacing was set to 0.375 Å, and the number of grid points in dimensions (x, y, z) was set to 70×70×70.

	Center coordinates (Å)		
**Protein**	**x**	**y**	**z**
HDAC 2	24.757	−16.735	0.794
HDAC 4	19.141	−8.645	−3.505
HDAC 8	36.691	12.592	124.207

Table S4. Bias coordinates applied to each target.

	Bias coordinates (Å)			V_{set}	r	type
**Protein**	x	y	z			
HDAC 2	19.975	−19.950	−2.076	−1.00	1.20	acc
	20.477	−17.980	−0.517	−1.00	1.20	acc
	29.134	−15.279	2.622	−1.50	1.20	don
HDAC 4	22.086	−13.364	2.450	−1.00	1.20	acc
	19.910	−12.172	1.778	−1.00	1.20	acc
HDAC 8	37.064	7.817	123.180	−1.50	1.20	acc
	36.949	9.063	120.977	−1.50	1.20	acc
	34.894	17.767	125.041	−0.50	1.20	aro
